# Influence of Working From Home During the COVID-19 Crisis and HR Practitioner Response

**DOI:** 10.3389/fpsyg.2021.710517

**Published:** 2021-09-23

**Authors:** Zhisheng Chen

**Affiliations:** College of Economics and Management, Nanjing University of Aeronautics and Astronautics, Nanjing, China

**Keywords:** working from home, COVID-19 crisis, influence, HR practitioner, response

## Abstract

The pandemic has changed the way people work, and more and more people are choosing to work from home (WFH). Unlike traditional work patterns, this approach has limitations and has had a significant impact on both organizations and individuals. It also brings many challenges to the work of HR practitioners. HR practitioners, as key players in strategic human resource management, need to take advantage of management innovations under the crisis to improve employees’ work flexibility and effectively address the impact of working from home. This study aims to address the need for employee skill improvement, psychological stress relief, work-family balance, and company culture reinforcement from an HRM perspective because of the impact of WFH work patterns during the COVID-19 crisis.

## Introduction

The recent rapid worldwide spread of a novel coronavirus infection (COVID-19 virus) has led to a severe global economic downturn (Al-Mansour and Al-Ajmi, 2020). Governments imposed total lockdown, banning non-essential travel, and requiring the closure of all non-essential activities. The strict government control measures led to many inconvenient working conditions. Traditional ways of working encountered serious challenges. The impact of COVID-19 on the global economy was similar to that of the 2008 crisis, although its long-term consequences were more severe. The impact on company performance is more pronounced in heavily impacted areas and industries, such as education and healthcare. We need to adopt a temporary COVID-19 strategy with companies responding quickly (Ahlstrom and Wang, 2020). Many companies have opted for flexible work practices, such as working from home to reduce the spread of disease and losses. During the COVID-19 crisis, most people were already using online commerce as well as work from home (WFH) and digital businesses. In response to the outbreak of the crisis, work patterns changed and the WFH model grew rapidly (Zou et al., 2020b). However, with the rise of WFH, its corresponding side effects emerged.

First, unlike traditional office models, WFH requires people to learn new online office skills along with virtual work communication skills. There may also be unplanned virtual work sessions. In addition, working from home requires attention to the confidentiality of office data to prevent leakage. This also raises the need to adapt to the new office environment, and employees’ WFH skills need to be trained and strengthened.

Second, when working from home, people lack face-to-face communication with colleagues, and once problems arise at work, it is difficult to solve them quickly through online virtual communication. Online network communication to solve problems leads to increased psychological stress and anxiety. This is also extremely harmful to people’s mental health.

In addition, WFH leads to the occupation of family members’ space. With the new crown pneumonia epidemic, people tend to occupy family space for their own work needs. When people work at home, some family tasks, such as childcare or housework, need to be shared between them. This creates a conflict between family and work. The imbalance between work and family can negatively impact job productivity, and HR practitioners need to consider how to mitigate this conflict.

Even, once the office work style is abandoned, people tend to overlook the impact of company culture. Since working from home people can only communicate and work through the virtual space of the Internet, people tend to ignore the role of culture. In fact, in times of crisis, the effect of culture cannot be neglected, and HR practitioners should take various measures to guide the role of culture.

The study found that while WFH has some advantages during this phase, it also has different effects on people, such as conflicts with family from taking up home space, inability to adapt to telecommuting, and lack of support from leaders or co-workers, but the following four areas are the objective and rationale of this study’s discussion.

1. Employees who Working From Home (WFH) face a home-based work environment where they need to learn special office skills.
2. WFH can make people feel isolated and can also lead to psychological stress.
3. During an outbreak, home-based employees often face conflicts between caring for their families and working.
4. Home-based employees often ignore the potential incentives of culture under the COVID-19 crisis.

Therefore, based on the impact of WFH on the above four aspects, this study proposes corresponding support measures from the perspective of human resource management.

## Literature Review

### Person-Environment (P-E) Fit Theory

The theory of person-environment fit, first proposed by Lewin in 1951 (Kahana et al., 1980), considers the positive benefits of person-environment matching for individuals. People adapt to changes in their environment (e.g., those who choose to WFH due to a pandemic) and reap the greatest benefits, such as avoiding the risk of contracting COVID-19 (Chung-Yan, 2006). In the wake of the new coronavirus outbreak, many academics and HR practitioners have been thinking about how to adopt flexible work arrangements (FWAs), such as WFH, as a more appropriate way of working.

Different aspects of the P-E fit model have been extensively studied by several scholars, and the person-environment fit can be subdivided into the person-vocation fit (P-V fit), person-job fit (P-J fit), person-group fit (P-G fit), and person-organization fit (P-O fit). From the division of these four concepts, it can be seen that the fit between the individual and the work environment should not only be in harmony with the organization at the macro-level, but also harmony with the work team at the micro-level, and most importantly, with one’s work (Cable and Derue, 2002; Ahmad, 2012).

The P-V fit is like the P-J fit, but the difference between the two is that the P-V fit tends to be more of a professional skills match. Without the professional skills required for the job, individuals cannot fully adapt to the work environment and thus constrain themselves, and the specialized skills needed by WFH require home-based workers to develop appropriate job skills, such as office skills and communication skills. These skills are different from those required for office work. New training models (online virtual) and content need to be provided.

Person-job fit refers to the idea that people meet the needs of their work, but they also derive satisfaction from their work. When individual satisfaction is not achieved, anxiety, stress, and psychological breakdowns can occur.

Person-group fit focuses on the need for harmony between the team and the work environment, and between the individual and the team, in order to achieve optimal work results. Employees who WFH are far from face-to-face interaction in the office; they are more likely to communicate online. It is important to know how to build online virtual teams. However, they also face another challenge: the “family and work imbalance.” In fact, in the WFH model, people spend more time with their families (another fun and challenging “team”). How family relationships are managed is also a way to support WFH.

Person-organization fit is an indication of the alignment of individual and organizational goals. Specifically, it means that the individual and the organization are aligned in terms of culture and values. The degree of personal and organizational culture fit directly affects the performance of employees. The pandemic brings about a shift in the way people work as well as get used to working from home. But they often ignore the importance and role of culture. Organizations need to consider how to strengthen the role of culture under WFH.

Several studies have shown that there should be consistency between HRM practices and P-E fit, especially in the context of COVID-19. By integrating strategic HRM with P-E, HRM practices and policies support P-E fit and thus gain competitive advantage for the firm.

Hypothesis 1: The P-E fit model can be used to produce satisfactory results for organizations and individuals in a pandemic crisis. The application of the model can also explain and support HRM in addressing the impact of the WFH model on job skills, stress, family, and culture.

### Crisis Management During a Pandemic

A crisis is a situation that affects a company’s organizational sustainability, performance, and ultimately threatens its viability. Managers are concerned that crises can negatively affect different types of businesses at any time and place, as in the case of the new crown pandemic crisis. In the workplace, crisis management is an effective response to a crisis at work – discrimination, physical injury, emotional harm, or some type of natural disaster. Implementing crisis management requires managers to understand what people need and how they can help.

Crisis management can be a challenging task, especially in an organization’s human resources department (HRD). HRD plays an important strategic role in crisis management, yet it is rarely described and analyzed in the literature (Christina and Fotios, 2015). Indeed, from a practical perspective, crisis management has been a neglected area of HRM, despite the growing recognition of the impact of different crises on performance outcomes. It is recommended that an effective crisis management team (CMT) be established to address issues of concern throughout the organization (Blythe, 2004). As a member of the CMT, the HR director is responsible for providing leadership to the company and its employees during a crisis event (COVID-19).

From the perspective of HR managers, a crisis-driven HR strategy is more effective. They believe that it is much easier to manage employees during a crisis than to manage other resources. The design of crisis management processes requires a high level of strategic integration between job skills, stress relief, work-family balance, and corporate culture (Wang et al., 2009).

The approach to crisis management used by HR should be different from that used by other functions. HR leaders address organizational crises through crisis management preparedness, including improving job skills, balancing work and family, relieving psychological stress, and strengthening culture (Lockwood, 2005).

Hypothesis 2: COVID-19 has a serious impact on the survival of the company, and the crisis management awareness of HR practitioners can reduce the impact of the crisis in four aspects: work skill improvement, psychological stress relief, family-work balance, and cultural role.

## Impact of WFH on Enterprises, Employees, and HR Practitioners

### Impact on Businesses

The new crown pneumonia pandemic caused widespread devastation in countries around the world. Tens of millions of people were infected; the economy was in recession and many people lost their jobs. Governments implemented many controls. These measures slowed the spread of the epidemic and some businesses were severely damaged.

A number of studies on the impact of the COVID-19 crisis on companies can draw preliminary conclusions about crisis management in companies. Companies in all industries, large and small, had to adapt their business models to changing environmental conditions within a short period of time. New crown pneumonia affects all corporate characteristics, including working methods, corporate performance, and corporate culture. After quickly responding to the new crown pneumonia crisis, they made a series of strategic adjustments to ensure the survival of the business.

1. The problem faced by the company is that the skills of the employees are not sufficient for the WFH pattern. The company needs highly skilled employees to carry out their work, but the office work skills of the past are no longer sufficient to meet the company’s needs.
2. The psychological stress caused by WFH to employees has a negative impact on the achievement of corporate goals.
3. This conflict cannot be avoided due to the replacement of office space with home space. The company needs to consider sacrificing home space to meet the work needs of employees.
4. The role of corporate culture is significantly weakened by the loss of physical distance contact.

Some agile companies have adopted strategies that include flexible HRM policies and practices, which are effective in the short term, but in the present and post-COVID-19 era crisis impact, companies need to focus on diversity and long-term HR strategy research.

### Impact on Employees

During the COVID-19 pandemic, the homes of employees suddenly became the main place of economic activity. Many countries have used their homes as a buffer against economic downturns and have taken action to support this WFH (Jenkins and Smith, 2021). We argue that businesses and governments see housing as a supporting pillar for economic development. Even employers who offer work-at-home jobs can be seen as an effective way to deal with the epidemic. It takes everyone – managers, employees, and their families – to adapt.

Advances in technology have made it possible for people to WFH, and this has affected the way people, especially staff. It has also benefited some companies during the difficult times of the pandemic. Companies have adopted the WFH model, relying on modern technology to reduce the corresponding regulatory costs (White, 2019).

Work from home can improve performance due to its flexibility. Employees can decide when and where to work. Many employees are satisfied with the flexibility they get from the WFH model. Working from home can also improve performance because there are no interruptions, employees have fewer breaks, and there is no contact with co-workers (Garg and Rijst, 2015).

However, some people use their home as a free workplace that can be used inexpensively in an emergency (e.g., COVID-19) but neglects its function as a place to live. As a result, employees are faced with corresponding challenges and problems:

First, the model requires upgrading employees’ WFH work skills. This demand for WFH is driving the digitization of human work at an alarming rate due to the explosion of COVID-19 (Savi, 2020). Employees need to work and communicate online, which requires special skills, such as new office skills and online communication skills. However, some specific industries, such as low-skilled services, cannot adopt this model. In addition, network accessibility and online task suitability can affect the feasibility of the model.

Second, internal psychological stress. Research has shown that a lack of social support and the feeling of working alone can lead to loneliness (Rook, 1985), and also to stress (Liu and Guo, 2007). Some individuals are more stressed during a pandemic because they are unable to communicate their anxiety to others. In addition, with uncertainty about the future, such as layoffs, pay cuts, and bankruptcies, employees experience a serious increase in internal stress. Further studies also found that employees’ psychological stress also has a direct negative impact on hiring commitment (Ali and Kakakhel, 2013; Velnampy and Aravinthan, 2013).

Third, employees who WFH often have conflicts between taking care of their families and their jobs during an epidemic. Telecommuters work longer hours than those who work in formal offices, which is a major reason for work-family imbalance. Their work style is flexible so they have unlimited access to online offices (Song and Gao, 2020); this model also breaks down the boundaries between work and non-work. It reduces the company’s need for office space but increases the employees’ need for living space because of the need for extra rooms to WFH (Behrens et al., 2021). In addition, when some employees need to take care of their families, their families may stay at home while they are working (Kara et al., 2021). Employees expect a dedicated workspace at home with fewer distractions from family members, which is associated with a better work-family balance (Allen et al., 2021).

Finally, the role of culture is weakened. In WFH situations, the functions embodied in corporate culture are weakened, such as leadership culture and cooperative culture. The manager’s ability to control and supervise subordinates is also affected. In addition, managers may be concerned about the impact of working from home on contracts and employee reputation. Unlike traditional office work, this model reduces opportunities for intimate psychological interactions while reducing face-to-face communication. While information and communication technology (ICT) can facilitate online interaction and collaboration with colleagues, they lack the enthusiasm for face-to-face interaction, which is seen as key to developing closer social relationships (Vayre and Pignault, 2014). Failure to address the lack of interpersonal interaction can ultimately lead to employees feeling disconnected from the corporate culture and work environment (Marzban et al., 2021; Wilson, 2021).

### Impact on HR Practitioners

In the field of human resource management, it has long been recognized that employees feel frustrated and stressed in situations of danger or uncertainty, such as COVID-19 (Kumar et al., 2021). The stress caused by an outbreak can provide HRM practitioners with constructive insights to help them assess opportunities and developments in an environment of threat and uncertainty. As the company continues to adjust its HR policies and practices in the face of COVID-19, it will be crucial to understand how these outbreaks affect employee P-E fit, and how to address dangerous misfits. The pandemic has had a serious impact on HRM policies and practices in different industries. The prominent impact is manifested in a series of challenges for HR practitioners as a result of the shift in work patterns.

1. What training approaches and innovative training content do HRM adopt for the WFH model where work skills differ from the traditional office requirements of the past?
2. How can HRM consider mitigating the increased internal stress of home-based workers, which is damaging to both individual and organizational performance?
3. How can HRM consider developing a more reasonable work-family balance plan, given the negative impact of workplace conflicts with families?
4. As one of the important promoters of corporate culture, how can HR practitioners enhance the role of culture in the new work model?

Not surprisingly, the COVID-19 pandemic has forced HR professionals to rethink and redefine their roles as organizations begin to adapt to the way people work (Nutsubidze and Schmidt, 2021).

Hypothesis 3: Organizations may adopt the WFH work model in the event of a pandemic, but it presents four unprecedented challenges for companies, individuals, and HR practitioners. In the face of the crisis and challenges, HR management needs to respond and react accordingly.

## HR Practitioners’ Reactions to the Impact of WFH

The main impacts during WFH are job skill requirements, psychological stress, conflict with family, and WFH culture. HR practitioners need to provide feedback and strategies to these impacts.

### Upgrading WFH Skills: Innovative Training Content and Methods

During the pandemic, employees lost their motivation to advance in their careers because they were working from home too long. They needed to develop the knowledge and skills to thrive in their current environment. As remote workers become more interested in improving their capabilities, HR practitioners should take the lead in organizing relevant skills training to meet employees’ desire to learn and grow. Predictably, when employees WFH, they will be trained to improve their performance and support the company’s growth in the post-pandemic era (Caligiuri et al., 2020).

Countless practical examples show that HRM has developed strategies to overcome the disadvantages of the pandemic. These strategies, such as innovations in training methods and content, contribute to improving employees’ competencies, maintaining their motivation, and reducing their psychological stress (Gigauri, 2020; Zou et al., 2020a).

Human resource management increasingly draws inspiration from online virtual training designs. When working from home, employees can learn a wide range of skills through online courses (Hamouche, 2021). To meet the needs of their own continuous development in a pandemic situation, HR departments are conducting training needs surveys and coordinating with other departments to design online training courses. It is conceivable that online virtual classes in the future will become the new normal for training in the era of the new pneumonia crown (Alhat, 2020).

In addition to the usual vocational skills training, the ongoing crisis has created new training needs, such as ICT. In particular, ICT can also be used to train recruits in work and collaboration skills. Many companies now see the need to prepare for a long-term pandemic as standard work content decreases and the WFM model remains intact. Currently, improving employee skills through training is the most effective use of time (Hamouche, 2021). This is driving a growing demand for technology-driven training programs around the world.

Often, many employees must use computers and the Internet while working from home. Human resource practitioners value the need for information security training. Employees who WFH should receive basic training on cybersecurity hazards and how to effectively prevent information risks and avoid leaks of private company data.

### Alleviate Psychological Stress Caused by WFM

According to the principle of personal preference in economics, not everyone wants to WFH (Perrigino and Raveendhran, 2020). The work-family conflict caused by WFH is a major source of stress for employees and has a negative impact on their psychological health (Sharma et al., 2016); this imbalance can lead to managers’ negative perception of WFH and affect their beliefs; and pressure from professional groups can also affect HR practitioners’ idea of working from home. HR practitioners can take some appropriate measures to alleviate this stress.

Psychologists believe that a home-friendly workplace can help reduce employee stress and depressive symptoms (Shepherd-Banigan et al., 2016). Based on a cost–benefit analysis, we believe that improved work-family relationships can influence organizational goals, such as improved organizational performance and corporate reputation. HR practitioners should play an important role in establishing such a workplace. Although employees WFH, HR practitioners can develop family-friendly work programs, interact with employees and their families, and show concern for employees during the COVID-19 crisis. The program usually consists of a “supportive family-friendly program” and a “supportive family management team,” which represents HRM efforts to help employees balance work and family responsibilities (Thomas and Ganster, 1995).

HR practitioners can provide stress relief training, such as stressor analysis, threat and infection risk prevention, mental health in WFH, and work-family balance. To reduce the risk of infection in face-to-face training, HR can provide online virtual training.

In addition, the sustainability of HRM policies and practices plays an important role in reducing employee dissatisfaction and job stress. Important HRM policies related to employees include compensation and benefits, performance evaluation, promotion, and transfer. With the impact of the pandemic, the sustainability of these policies helped to alleviate the mental stress and job slack of the work-at-home employees.

### One Support: Work-Family Balancing

Some companies affected by new coronary pneumonia have had to adopt flexible working practices to reduce the risk of an outbreak. According to various analyses, WFH can help employees balance career development and work. It can also reduce commuting time and companies can save on office costs by having smaller office spaces (Gajendran and Harrison, 2007). However, WFH has a significant impact on the work-home balance (Perrigino and Raveendhran, 2020). WFH often blurs the boundaries between work and home roles (Schieman and Young, 2010). Even people who WFH are not satisfied with the distribution of family tasks after working for a long period of time. Long working hours and occupying home space also increase the likelihood of work–family conflict (Solís, 2016). We believe that helping employees achieve work-life balance should be a key part of HR practices and strategies if it ensures optimal employee satisfaction, rather than leaving them feeling dissatisfied, exhausted, and stressed.

Research has found that work–family conflict is more correlated with work schedule flexibility when working from home. In fact, WFH is divided into traditional (typical “9–5” working hours) and non-traditional (irregular working hours; Duxbury et al., 1996). Non-traditional WFH has a high degree of flexibility in terms of work schedule, which is different from traditional work hours. When developing WFH policies, HR practitioners should fully consider the individual preferences of WFH employees, based on the different characteristics of WFH employees (Golden, 2012). Based on individual preferences, HR policies should allow telecommuters to flexibly schedule their working hours to meet their individual needs. Flexible work schedules for non-traditional WFH can alleviate work–family conflicts during a pandemic.

In addition, the potential for work–family conflict increases with longer work hours. During the COVID-19 pandemic, telecommuters used their home space as an office. Unlike traditional office work, they spent more time on work without realizing it (Dockery and Bawa, 2020). The relatively less time spent with family members undoubtedly leads to strong work-at-home conflicts. Even some telecommuters work hard on weekends and holidays. Long hours of WFH can increase family unhappiness and stress, which can affect the family atmosphere (Song and Gao, 2018). HR practitioners should consider a policy of not working long hours when developing work schedules. An effective HR policy can alleviate the excessive work hours caused by remote workers who work too many hours at home.

Finally, the assignment of family tasks is particularly important for home-based work balance. Indicators of family functioning include perceptions of the quality of relationships and the degree of equity in how family tasks are shared within the family. These indicators of family functioning are used to indicate the extent of work–family conflict (Bankwest Curtin Economics Centre, Curtin University School of Economics et al., 2017). Family members will perceive that the division of responsibilities within the family is fair and reasonable, and rationality will influence the relationships between family members. Companies, especially human resource practitioners, should provide employment assistance programs for remote workers during the pandemic, including family member relationship management, reconciliation of family and work responsibilities, and post-conflict resolution.

### Cultural Reinforcement in the WFH Model

Corporate culture is a complex network of corporate norms, organizational vision, and member attitudes with specific group characteristics. It can be reinforced through training, punishment, rewards, etc. (Kuchinke, 1999). Culture needs to be adapted to the environment external to the organization, especially in the context of the new crown pandemic crisis. As the impact of the pandemic crisis expands and the psychological stress of work-at-home employees increases, corporate culture will become a focus of attention for managers or HR practitioners.

If HR practitioners want to reduce the disruption of WFH and improve WFH arrangements, they should create a WFH culture with high execution and low barriers. HR practitioners need to focus on the following areas:

When selecting and hiring talent during a pandemic, HR needs to focus on whether these individuals are a good fit for the company culture. P-E fit theory states that individuals will choose companies when their ideas align with the organization’s culture, WFM model, and development philosophy. Newly hired employees still need to receive initial culture training from the company.

Because of the pandemic, working from home requires a more collaborative spirit. Remote workers enhance communication and collaboration with each other through virtual organizations. Despite their different backgrounds and lack of face-to-face interaction, a collaborative culture is more likely to emerge in the WFH model (Borkovich and Skovira, 2020; Singh and Kumar, 2020).

Research has shown that leadership is a culture-specific element. HR practitioners should have some leadership in times of crisis to help their companies survive the crisis. We believe that HR practitioners with strong leadership skills are able to identify and effectively deal with cultural conflicts in different work models. Several studies have concluded that leadership culture in crisis enables employees to successfully deal with the effects of crisis (Colville and Murphy, 2006). In the current work model, managers with leadership skills are able to meet employees’ expectations and guide the company through the effects of the pandemic.

An overly idealized employee culture, such as high performance and high results orientation, can lead to cultural barriers during WFH. The perception is that leaders want employees to work properly in the office, and when they WFH, managers cannot directly supervise them or communicate face-to-face about their performance. This is why employees do not want to WFH (Lott and Abendroth, 2019). HR practitioners should encourage managers to rationally evaluate the performance of employees who WFH through an online evaluation system. In addition, work-from-home support programs for HR practitioners can help reduce the cultural barriers in the WFH model.

## Future Research Direction

Work from home will continue to have an impact on business and work. In addition to the recommendations, the study has already provided, there are some theoretical and practical studies on the HR side that need to be strengthened in the current and post-pandemic era: Organizations use E-HR in the WFH model to help HR practitioners work more efficiently; during the pandemic, traditional offline HR operations were replaced by online work. Virtual online HR management is required to complete recruitment and staffing; unlike office workflow, HR practitioners need to optimize job responsibilities and workflow for employees working from home; and the impact of the crisis has led to a more popular relationship-oriented HR system. This system brings employees tighter together with the organization and brings a strong organizational commitment; in a pandemic situation, a period HR strategy should be developed to help the organization overcome the crisis; and corporate social responsibility helps companies to improve performance and better position themselves during the pandemic.

## Limitations

The study needs to strengthen the literature citations. Though the study reviews a range of literature and what we have reviewed is current and relevant, the recommendations need to be bolstered by the previous literature. In addition, this paper only analyzes the effects of WFH on people during COVID-19 and should compare the effect before the COVID-19 outbreak. Therefore, the study needs to compare and analyze the influence from the timeline.

In the future, we also need to consider: Does WFH still widespread in the post-COVID-19 era? If there are still many companies using the WFH model, what is the impact on people at that time?

## Conclusion

COVID-19 has affected the lives of many people. To prevent the future spread of this pandemic, many organizations have had to change their traditional ways of working. The advent of WFH has brought some convenience, but as the impact of the pandemic has deepened, it has also had certain effects on organizations, employees, and HR practitioners that will continue in the post-pandemic era. Through a discussion of the relevant literature, this study argues that during the COVID-19 crisis, when people were working from home, our managers, especially HR practitioners, needed to address issues, such as job skills enhancement, employee stress under the crisis, work-family imbalance, and corporate culture reinforcement. Focusing on these issues has benefits for both organizations and individuals, especially in the current crisis. Future research will also need to consider the implications of this work model in the post-COVID-19 era.

## Author Contributions

ZC contributed to conception and design of the study. He also contributed to manuscript revision, read, and approved the submitted version.

## Conflict of Interest

The author declares that the research was conducted in the absence of any commercial or financial relationships that could be construed as a potential conflict of interest.

## Publisher’s Note

All claims expressed in this article are solely those of the authors and do not necessarily represent those of their affiliated organizations, or those of the publisher, the editors and the reviewers. Any product that may be evaluated in this article, or claim that may be made by its manufacturer, is not guaranteed or endorsed by the publisher.
